# Association of Polycystic Ovarian Syndrome (PCOS) With Vaginal Microbiome Dysbiosis: A Scoping Review

**DOI:** 10.7759/cureus.62611

**Published:** 2024-06-18

**Authors:** Maria P Pereira, Sydney Jones, Joshua M Costin

**Affiliations:** 1 Department of Obstetrics and Gynecology, Dr. Kiran C. Patel College of Allopathic Medicine, Nova Southeastern University, Fort Lauderdale, USA; 2 Department of Medical Education, Dr. Kiran C. Patel College of Allopathic Medicine, Nova Southeastern University, Fort Lauderdale, USA

**Keywords:** streptococcus, women’s health, vaginal microbiota, lactobacillus, vaginal dysbiosis, polycystic ovaries, vaginal microbiome, polycystic ovary syndrome (pcos)

## Abstract

The aim of this scoping review was to explore the potential relationship between vaginal microbiome dysbiosis and polycystic ovarian syndrome (PCOS). Four databases were utilized to identify primary literature based on a pre-determined exclusion and inclusion criteria. The electronic databases searched include MEDLINE, Embase, Cochrane Register of Controlled Trials (CENTRAL), Cumulative Index to Nursing and Allied Health Literature (CINAHL), and Web of Science. After an initial double-blind screening and removal of duplicates, 81 articles remained. Articles were included based on preselected inclusion and exclusion criteria, type of study, and date of publishing. Specifically, primary literature that focused on subjects that were diagnosed with PCOS and that discussed PCOS in relation to the vaginal microbiome was included. Literature reviews, studies with animal subjects, and studies that did not discuss PCOS and the vaginal microbiome were excluded.

Current data from the five articles included in this review suggests that there is a relationship between PCOS and vaginal microbiome dysbiosis. Specifically, dysbiosis of the vaginal flora may be due to vaginal pH alterations secondary to decreased vaginal *Lactobacillus* species and elevated pathogenic species including *Streptococcus, Actinomyces, Prevotella, Gardnerella*, and *Mycoplasma* species. The manifestation of this vaginal microbiome dysbiosis is often bacterial and fungal vaginitis. Therefore, more studies are needed to explore the possibility of treating PCOS with probiotics designed to reestablish a healthy *Lactobacillus*-dominant vaginal microbiome. In addition, further studies on the microbial composition of the vaginal microbiota in PCOS patients could identify microbial biomarkers for diagnosing PCOS.

## Introduction and background

Polycystic ovarian syndrome (PCOS) is the most common endocrine disorder affecting women of reproductive age, with a prevalence between 5% and 16%, depending on the patient population and criteria used for diagnosis [1,2]. It is characterized by polycystic ovaries, amenorrhea or oligomenorrhea, hirsutism, acne, and insulin resistance. Menstrual irregularities in PCOS are proposed to be of multifactorial etiology, including intra-ovarian factors, diminished follicle-stimulating hormone (FSH) levels, and aberrant gonadotropin regulation. Ultimately, these factors lead to the arrest of antral follicle development and therefore decreased or absent ovulation, resulting in oligomenorrhea or amenorrhea, respectively [3]. In many cases, the unpredictability or absence of ovulation leads to infertility or difficulty conceiving. In fact, PCOS has been implicated as the primary cause of 70% of anovulatory infertility cases seen in young women [4]. Although the exact etiology of PCOS is unknown, the hyperandrogenism seen in the condition is hypothesized to be in part due to testosterone-overproducing ovarian theca cells in PCOS patients. This overproduction of androgens may be due to elevated luteinizing hormone (LH) levels seen in PCOS. Although the role of LH is to regulate androgenic production of theca cells, it is important to note that FSH regulates the aromatase activity of granulosa cells. In other words, hyperandrogenism may also be in part due to low levels of FSH preventing granulosa cells from converting excess androgenic precursors to estrogen [1]. Androgen excess may manifest as hirsutism, acne, and male pattern hair loss [1,5]. Although there is no cure, PCOS is typically treated with prescribed oral contraceptives to regulate the menstrual cycle, metformin to decrease insulin resistance, and/or spironolactone to treat symptoms derived from the presence of androgen excess [1].

The vaginal and gut microbiomes are defined as the communities of microorganisms that reside in the vagina and gut, respectively. The vaginal microbiome is very dynamic in nature and dysbiosis can arise due to various factors that lead to an altered vaginal pH or abnormal ratios of reproductive hormones. Such factors include gestational status, menstrual cycle, sexual activity, and contraceptive use [6,7]. In the context of PCOS, an aberrant LH to FSH ratio likely contributes to decreased *Lactobacillus* in the vaginal microbiome, therefore leading to dysbiosis [8]. This is significant as these *Lactobacillus* species serve to lower the vaginal pH, which is needed to create an unfavorable environment for the growth of pathogenic species such as *Candida albicans* [9]. In contrast, a non-*Lactobacillus*-dominated vaginal microbiome is associated with an increased risk of sexually transmitted infections and obstetric complications [10]. Though it is common for a woman to suffer from at least one episode of vulvovaginal candidiasis during their lifetime, the prevalence of vulvovaginal candidiasis can be as high as 13.5% in PCOS patients [11].

Likewise, the gut microbiome has an important immunomodulatory function, which can be pathogenically altered in obese patients with a high-fat-low-fiber diet [12]. It is currently hypothesized that gut microbiome dysbiosis in the previously mentioned population activates the immune system and ultimately leads to insulin receptor malfunction and hyperinsulinemia. Hyperinsulinemia is believed to increase ovarian androgen production and aberrantly affect antral follicle development. This hypothesis could potentially explain the pathophysiology of PCOS, as it accounts for the hyper-insulinemic, hyperandrogenic, and anovulatory states seen in PCOS [12]. Furthermore, crosstalk exists between the gut and vaginal microbiomes. For instance, although the short-chain fatty acids in the gut microbiome contribute to a state of homeostasis and promote eubiosis in the gut, they may lead to inflammation and dysbiosis in the vagina [13]. Although the literature has explored the role of the gut microbiome in PCOS, the development of vaginal microbiome dysbiosis in PCOS has not been researched thoroughly. In turn, the aim of this review is to explore the relationship between vaginal microbiome dysbiosis and PCOS and identify potential theories for the mechanism of action for this relationship.

## Review

Methods

Eligibility Criteria 

The study was carried out utilizing the Joanna Briggs Institute (JBI) guidelines for scoping reviews. Inclusion and exclusion criteria were defined to ensure there was a sound method to assess study eligibility. Inclusion and exclusion criteria are outlined in Table 1. Primary literature studies whose subjects were patients diagnosed with PCOS, studies that took place between 2011 and 2022, and studies that discussed PCOS and the vaginal microbiome were included in the review, while meta-analyses, reviews, and editorials were excluded. Women who were pregnant, not assigned female at birth, or menopausal were not included since these individuals would have significantly altered hormones compared to premenopausal, non-pregnant PCOS patients, which would affect the vaginal microbiome as well as the presentation of PCOS itself. Furthermore, animal studies were excluded to generate results more applicable to human patients.

**Table 1:** Inclusion and Exclusion Criteria PCOS, polycystic ovarian syndrome

Study characteristics	Inclusion criteria	Exclusion criteria
Study design	Primary literature (randomized controlled trials, cohort, case-control, case reports, cross-sectional)	Literature reviews, scoping reviews, meta-analyses, editorials
Population	Subjects with PCOS, assigned female at birth, premenopausal, non-pregnant, of any ethnicity	Animals, pregnant, or menopausal individuals
Outcomes	Discusses PCOS and vaginal microbiome	Does not discuss PCOS and vaginal microbiome
Publication year	2011-2022	Before 2011

Strategy

The population, concept, and context (PCC) strategy was utilized in Table 2 as a framework to formulate and then pinpoint the main concepts to be addressed by the research question: "Is there an association between PCOS and vaginal microbiome dysbiosis?” Searches were conducted in MEDLINE, Embase, Cochrane Register of Controlled Trials (CENTRAL), Cumulative Index to Nursing and Allied Health Literature (CINAHL), and Web of Science, on August 9, 2022. Search terms used were terms for vaginal microbiome combined with synonyms of PCOS. Specifically, the search terms used included ('polycystic ovar*' or 'ovar* polycystic' or 'pcos' or 'stein leventhal' or 'stein cohen leventhal' or 'sclerocystic ovar*' or 'micropolycystic ovar*' or 'ovar* syndrome') AND ('vagina* d?sbios*' or 'vagina* d?s symbiosis' or 'vagina* d?sbacterios*' or 'vagina* microbio*' or 'vagina* micro*' or 'vagina* flora' or 'vagina* bacteri*' or 'vagina* microflora' or vaginosis or vaginitis or 'vagina* infection' or 'genital infection' or 'cervicovagina* microbio*' or 'genital tract microbio*' or 'cervical microbio*'). The search terms were developed by an instruction librarian in collaboration with one of the authors (MP). Moreover, the PRISMA (Preferred Reporting Items for Systematic Reviews and Meta-Analyses) method was used in order to track progress in the study selection process.

 

**Table 2:** Population, Concept, and Context (PCC) Framework The population, concept, and context framework was conceptualized from which a research question was generated and the main goals were defined for the study. PCOS, polycystic ovarian syndrome

PCC Elements	Definition
Population	PCOS patients
Concept	Vaginal microbiome dysbiosis
Context	Research publications on or after 2011

Study Selection and Data Appraisal

Utilizing the search terms mentioned previously pertaining to the vaginal microbiome and PCOS, a search was conducted across five databases, which initially resulted in 138 articles being imported into Rayyan (www.rayyan.ai) for screening. Fifty-seven duplicate articles across databases were removed via Endnote (version 21, Clarivate Analytics, Philadelphia, PA), and the initial double-blind screening was conducted by two authors (MP and JMC) on the remaining 81 articles. The initial screening consisted of utilizing the established inclusion and exclusion criteria, as outlined in Table 1, in order to determine the study’s eligibility based on its title and abstract.

The secondary screening was done using the same eligibility criteria defined in Table 1, in which the articles were retrieved and read fully (MP and JMC). The resulting articles were then assessed for quality and five articles were ultimately included in the study.

A data-charting form was developed by two reviewers in order to determine which variables should be extracted (MP and JMC). One reviewer charted the data (MP), then both discussed the results, and continuously updated the data-charting form in a repetitive manner.

The five articles that were selected from the full-text reviews were evaluated for method quality and risk of bias using the JBI Critical Appraisal Tools. Three articles were evaluated using the JBI critical appraisal checklist for case-control studies, one article was evaluated using the JBI critical appraisal checklist for cohort studies, and one using the JBI critical appraisal checklist for analytical cross-sectional studies. Each article was evaluated by each author, and discrepancies between the scoring of the evaluators were discussed and resolved, with the three authors ultimately agreeing upon a checklist score. Studies were included if they had a low risk of bias or better, defined as a score of over 70% following JBI critical appraisal method. All five articles were included and had a low risk of bias (mean score of 92%) according to the JBI checklists.

Results

**Figure 1:**
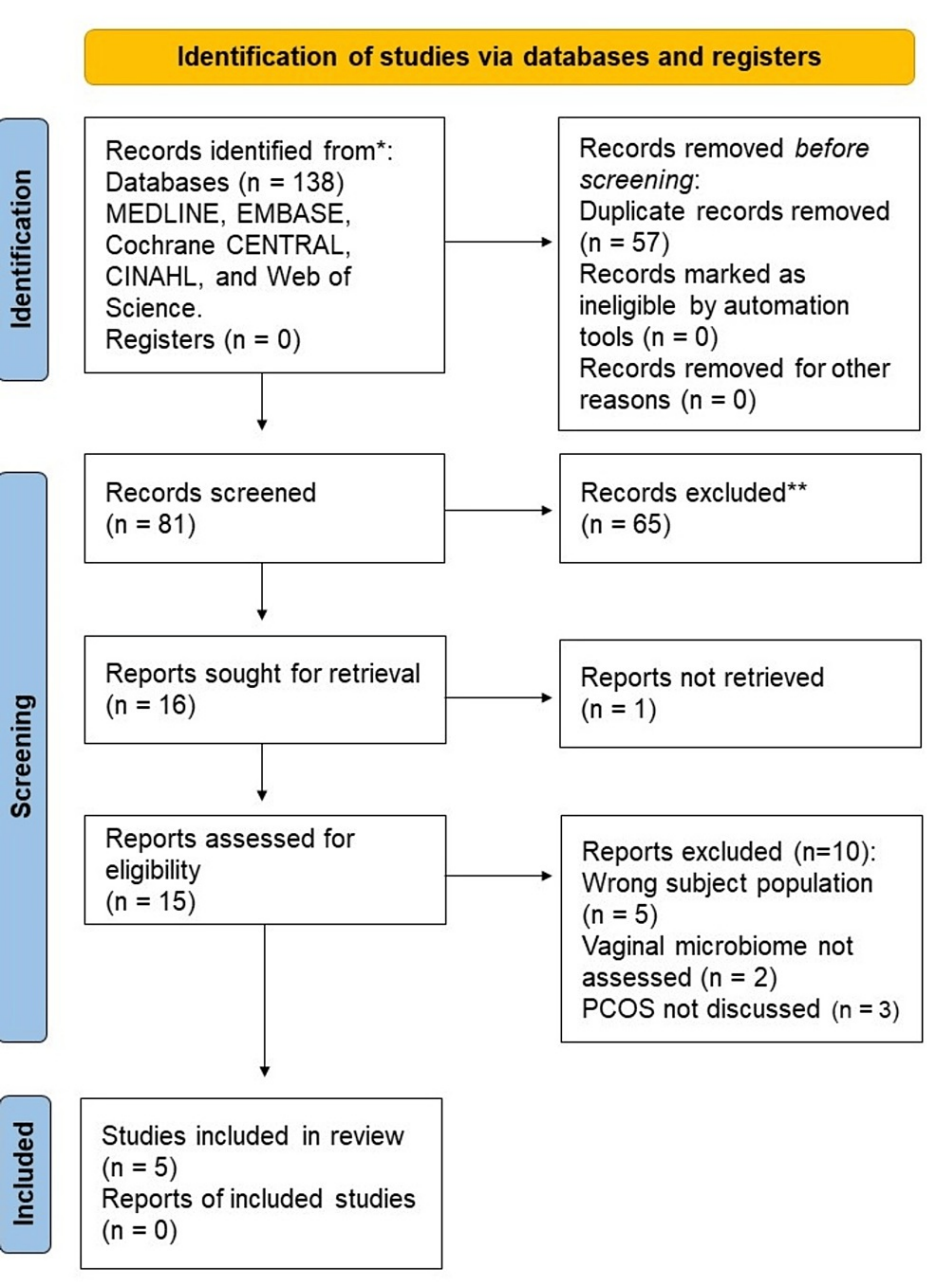
Preferred Reporting Items for Systematic Reviews and Meta-Analyses (PRISMA) Diagram. Joanna Briggs Institute (JBI) standard flow chart documenting the article screening process.

The initial database search returned 138 articles with five articles meeting all inclusion and exclusion criteria to be included in the study (Figure 1). The data was extracted and charted in Table 3. Across three studies, vaginal *Streptococcus *species and their role in reproductive hormone changes was explored. One study showed that *Streptococcus *was negatively correlated with serum FSH levels [14]. Another study suggested that an increase in *Streptococcus* species is associated with intermenstrual bleeding [11]. In addition, almost all the studies except for Lu et al. (2021) determined that *Mycoplasma* species were elevated in the vaginal microbiome of women with PCOS. Using *Mycoplasma *as a potential biomarker for PCOS was proposed as this species was found to be significantly elevated in PCOS patients when compared to a healthy control [14]. Similarly, one particular study postulated *Actinomyces* as a future potential biomarker in the context of PCOS diagnosis [14].

**Table 3:** Data Table. The studies chosen for the current review and the data extracted from each of them are shown. Species listed refer to levels in PCOS patients when compared to healthy controls without PCOS.

Study	Year	Study design	PCOS diagnosis method	Lower *Lactobacillus* genus	Lower *Lactobacillus* species	Elevated interest species
Hong et al. [6]	2020	Case-control	Rotterdam criteria	Yes	L. crispatus	*Mycoplasma *and *Prevotella*
Tu et al. [8]	2020	Case-control	Rotterdam criteria	Yes	*Lactobacillus *genus. Species not specified	*Gardnerella*, *Prevotella*, *Mycoplasma hominis*, *Streptococcus*
Hong et al. [11]	2021	Cross-sectional	Rotterdam criteria	Yes	*L. crispatus*, *L. iners*	*Gardnerella*, *Mycoplasma*, *Prevotella*, *Streptococcus*
Lu et al. [14]	2021	Case-control	Rotterdam criteria	Yes	*Lactobacillus *species not specified	*Actinomyces*, *Streptococcus*
Salah et al. [15]	2013	Cohort study	Rotterdam criteria	Yes	Species not specified	*Gardnerella *and *Mycoplasma*

Discussion

This scoping review identified five primary peer-reviewed articles on the vaginal microbiome in patients with PCOS. Although more papers pertaining to PCOS have been published in the last decade, the focus has remained on the disorder’s relationship to infertility and the gut microbiome. Based on our findings, there are a limited number of papers discussing the vaginal microbiome in relation to PCOS. The existing literature reviewed in this study, however, explored this relationship and suggested a higher incidence of vaginal microbiome dysbiosis in patients with PCOS than in healthy patients. In these studies, vaginal microbiome dysbiosis was associated with multiple conditions, including bacterial vaginosis, inflammation, insulin resistance, infertility, hirsutism, and acne. As these conditions are common clinical manifestations of PCOS, these findings may suggest that dysbiosis of the vaginal microbiome may play a role in the pathogenesis of PCOS and maintaining a healthy vaginal microbiome could improve symptoms and progression of the disease. For instance, it is possible than an aberrant vaginal microbiome may disrupt immune homeostasis in the female genital tract and allow for the production of pro-inflammatory cytokines that contribute to the pro-inflammatory state seen in PCOS [16].

Vaginal Lactobacillus Species

The five peer-reviewed studies that met the determined inclusion and exclusion criteria noted significant decreases in *Lactobacillus *species in patients with PCOS. *Lactobacillus *species are crucial for maintaining a healthy vaginal microbiome. Through the process of homolactic fermentation, *Lactobacillus* species process glycogen into various biochemical products including lactic acid [17]. The lactic acid produced by the *Lactobacillus *species is responsible for maintaining the acidic vaginal pH at 3.5-4.5, which creates an environment that is unfavorable for the growth or proliferation of harmful bacteria, viruses, and parasites [17].

The most common *Lactobacillus *species comprising the vaginal microbiome are *L. crispatus*, *L. iners*, and *L. gasseri*. After adjusting for possible confounding variables, such as body mass index (BMI), vaginal hygiene, and age, one of the studies noted a decrease in *L. crispatus* species in PCOS patients when compared to the control group [6]. Like other *Lactobacillus *species, *L. crispatus* prevents vaginal colonization by pathogenic bacteria and fungi. It does this not only by contributing to the lowering of the vaginal pH but also by producing antimicrobial peptides and bacteriocins, which further reduce the risk of pathological vaginal colonization. Similarly, another study recognized an association between *Lactobacillus* species dominance and a lower risk of bacterial vaginosis and sexually transmitted infections [8]. One more study noted a positive correlation between *Lactobacillus* species and FSH levels as well as a negative correlation between low FSH levels and increase in vaginal opportunistic pathogens. The reduction of *Lactobacillus *species in the vaginal microbiome has, therefore, been implicated as a risk factor for developing vaginal infections. These findings can explain why women with PCOS have a higher risk of bacterial vaginosis and vulvovaginal candidiasis compared to healthy patients [11,16].

Although research supports the idea that a vaginal microbiome with decreased *Lactobacillus *species increases the risk of vaginal infections, it is not fully understood what causes the decrease of *Lactobacillus *species in the first place. However, current literature has proposed that hormone imbalances, such as those seen in menopause and post-partum period, are associated with vaginal microbiome dysbiosis [6]. Similarly, other studies have suggested that vaginal microbiome dysbiosis may increase the production of local inflammatory factors, including tumor necrosis factor-alpha and interleukin-8, which may affect ovarian function and therefore influence the hypothalamic-pituitary-ovarian axis [11,18]. Another proposed pathway presents that changes in sex hormone levels reduce vaginal mucosal immunity, because sex hormones modulate certain aspects of mucosal immunity, including immunoglobulin production, T cell activity, and cytokine production [11,19]. This, in turn, may negatively affect vaginal microbiome stability. Like testosterone, estrogen level has also been found to modify the vaginal microbiome. Estrogen causes glycogen to accumulate in the vaginal epithelium, which is utilized by microbiome-dominating *Lactobacillus* species in homolactic fermentation [11,19,20]. Potentially, a decrease in estrogen could reduce *Lactobacillus* species. In the context of PCOS, estrogen may be elevated due to aromatization of androgens to estrogen. As a result, a decrease in *Lactobacillus* in women with PCOS appears more likely to be attributed to a different mechanism. It is possible that the interplay between the gut microbiome and sex hormones may impact the composition of the vaginal microbiome [11]. Overall, the association between hormone levels and the vaginal microbiome is multifactorial, likely involving multiple mechanisms, and more research is necessary to understand this relationship better.

Vaginal Pathogenic Species

Although *Lactobacillus* species were found to be decreased in the vaginal microbiome of patients with PCOS, current literature also indicates a greater abundance of other microbes in these patients compared to healthy controls, including *Mycoplasma*, *Prevotella*, *Actinomyces*, *Gardnerella*, and *Streptococcus *species (Table 3). Three studies indicated a significantly higher prevalence of *Mycoplasma *in patients with PCOS than in healthy patients [6,8,11]. The most common *Mycoplasma *species inhabiting the vagina is *M. hominis*, a Gram-negative opportunistic non-motile bacterium lacking a cell wall [21]. The overabundance of this bacterium has been specially observed in patients with bacterial vaginosis [11]. Bacterial vaginosis is positively correlated with infertility in patients with PCOS [15]. Although the main cause of infertility in this population has been shown to be anovulatory cycles, bacterial vaginosis may be an additional contributory factor when it comes to infertility in PCOS patients. Moreover, one study indicated that *Mycoplasma *was the most distinguished genus in PCOS, and the presence of this species could be a potential biomarker for PCOS screening. In this study, women with a relative abundance of more than 0.02% of *Mycoplasma* in the vaginal microbiome were at high likelihood of having PCOS [6]. Further investigation should be undertaken to determine the clinical effectiveness of utilizing *Mycoplasma *screening as a biomarker for diagnosing patients with PCOS.

*Prevotella* was also found in significantly greater abundance in PCOS patients’ vaginal microbiome in three of the studies [6,8,11]. *Prevotella *is an anaerobic bacterium that utilizes mucin-degrading enzymes, including sialidases and glycosidases, that negatively impact the vaginal mucosa and result in increased vaginal inflammation, which facilitates the growth of other pathogenic bacteria [11]. One study proposed that high levels of testosterone in PCOS can increase glycogen production, raising the pH and making the environment more vulnerable to infiltration by other pathogens [11]. This increases the risk of developing bacterial vaginosis, pelvic inflammatory disease, and infertility.

*Actinomyces *species were found to be significantly more abundant in non-*Lactobacillus*-dominated PCOS patients in comparison to *Lactobacillus*-dominated healthy patients [14]. *Actinomyces *species are Gram-positive anaerobic-to-microaerophilic commensal bacteria that have also been associated with bacterial vaginosis. This species has been promoted as a potential biomarker; however, a larger sample size would be required to verify *Actinomyces *as a predictive measure [14].

Three of the peer-reviewed studies noted an elevated abundance of *Gardnerella *species in the vaginal microbiome of patients with PCOS [8,11,15]. *Gardnerella vaginalis*, the causative agent of bacterial vaginosis, is a Gram-variable facultative anaerobic bacteria that may be introduced to the vaginal microbiota via sexual intercourse [22]. One study suggested that the increase in *Gardnerella *species witnessed in PCOS patients’ vaginal microbiome may be the primary contributor to the higher incidence of bacterial vaginosis seen in these women [8] As *Lactobacillus *species decrease, the vaginal pH increases, allowing for the overgrowth of anaerobic bacteria such as *Gardnerella*. *G. vaginalis* subsequently perpetuates a decreased vaginal immune response by competing with *Lactobacillus* species, producing a biofilm community and vaginal mucin-degrading enzymes, such as sialidase and vaginolysin [22,23]. Not only did PCOS patients have a higher incidence of bacterial vaginosis than healthy controls, but patients with PCOS with infertility had a higher prevalence of bacterial vaginosis when compared to PCOS patients without infertility [15]. Indirect actions of *Gardnerella *may contribute to infertility, including reducing vaginal immunoprotective mechanisms and allowing for coinfection with pelvic inflammatory disease-causing pathogens, such as *Chlamydia *and *Gonorrhea* [24]. One study showed that an abundance of *Gardnerella* was positively correlated with PCOS severity and negatively correlated with serum FSH levels [11]. These findings suggest a potential relationship between the abundance of *Gardnerella* and PCOS pathogenesis by contributing to decreased vaginal immunity. It is possible that increased susceptibility to vaginal infections lead to increased production of pro- inflammatory cytokines, subsequent ovarian inflammation, and ultimate ovarian dysfunction. This is under the assumption that *Gardnerella *decreases vaginal immunity enough to enable other pathogens to cause pelvic inflammatory disease. However, more research needs to be done in this area to further explore *Gardnerella*’s role in PCOS.

Lastly, *Streptococcus* species were also found by three studies to be significantly increased in patients with PCOS [6,11,14]. Specifically, dysbiosis in the vaginal microbiota creates a favorable environment for *Streptococcus agalactiae*, also known as Group B *Streptococcus* (GBS). *Streptococcus*’ negative correlation with FSH level and menstrual cycle regularity supports the hypothesis that *Streptococcus* influences sex hormone levels and may be implicated in ovarian dysfunction in PCOS.

Although all the species just listed are considered pathogenic in the context of the vaginal microbiome, *S. agalactiae* is a species that may have more serious implications pertaining to neonatal meningitis, sepsis, and pneumonia. Unfortunately, a gap in the literature exists regarding the incidence of vaginal *S. agalactiae* in pregnant women with PCOS. It is important that future research is done in this area with the aim of exploring if there is an increase in the incidence of vaginal *S. agalactiae *in this specific patient population that would be clinically significant enough to require earlier screening of vaginal *S. agalactiae* colonization.

Study Limitations

This scoping review provides a comprehensive overview of the existing literature on PCOS and the vaginal microbiome. However, one notable limitation was the limited number of included articles. Although it is possible that this is due to there being a limited amount of research on this topic, it is also indicative of the limited number of databases that were used to look for articles. Future research is necessary to fully understand the connection between PCOS pathogenesis and the vaginal microbiome. Moreover, the exclusion of studies involving pregnant and menopausal patients might have restricted the generalizability of the findings. Future iterations should consider further investigation into specific species within the vaginal microbiome as well as broadening the patient population to evaluate unique considerations for pregnant and menopausal patients.

Among the five papers included in the scoping review, some limitations were identified. Three of the studies included a small sample size, reducing their generalizability [6,11,14]. One study admitted to not measuring the absolute vaginal microbiome, instead relying on the relative microbiota abundance as it was more practical but still significant [6]. The same study also did not consider some confounding variables that have the potential to alter the vaginal microbiome, such as contraception methods, vaginal lubricant use, and pH of the vaginal samples [6]. Similarly, another study omitted important confounding variables, such as ovulation rate and pregnancy rate, which were felt to be relevant in consideration of FSH [14]. Although it included a large sample size, the comparative analysis of Tu et al. lacked confounding variables as well, such as hormone levels, menopause, age, and hygienic habits [8]. Additionally, the study excluded patients presenting with vaginitis, which may have subsequently excluded many PCOS patients, considering a positive association between PCOS and vaginitis has been established [8].

Two of the studies had limitations due to the nature of the study [15,11]. The cross-sectional design of one study limited the ability to infer causal associations, especially considering a single sample is not representative of a dynamic vaginal microbiome [11]. The vaginal microenvironment was also not evaluated utilizing the standard Nugent score, which limits the comparability of the data to relevant literature [11]. In one study, randomization of participants was not possible as patients refused participation in the control sub-groups [15]. Additionally, in this study, there was a lack of follow-up to test for bacterial vaginosis recurrence or resistance following treatment [15].

## Conclusions

 

This review aimed to explore current literature to determine if there was an association between vaginal microbiome dysbiosis and PCOS. Although the existing evidence is limited, current data does point towards an association between PCOS and vaginal dysbiosis. PCOS appears to be associated with a vaginal microbiota dysbiosis. Specifically, PCOS appears to be associated with a vaginal microbiota characterized by decreased *Lactobacillus* species and elevated pathogenic species such as *Mycoplasma *and *Streptococcus*. Future studies are needed to explore the clinical significance of an increased incidence of vaginal *S. agalactiae* in pregnant PCOS patients, as this specific microorganism can have significant health repercussions in neonates. In addition, further studies on the microbial composition of the vaginal microbiota in PCOS patients could identify microbial biomarkers for diagnosing PCOS and provide further insight into this topic.
